# Laser ultrasound wave pattern analysis for efficient defect detection in samples with curved surfaces

**DOI:** 10.1016/j.pacs.2024.100654

**Published:** 2024-09-27

**Authors:** Markus Saurer, Guenther Paltauf, Robert Nuster

**Affiliations:** Department of Physics, University of Graz, Universitaetsplatz 5, Graz, 8010, Austria

**Keywords:** Laser ultrasound, Finite curved samples, EFIT simulation, Welding seams, Ultrasound wave fields, Industrial applications, Efficient defect detection

## Abstract

Many production processes involve curved sample surfaces, such as welding or additive manufacturing. These pose new challenges to characterization methods for quality inspection, which are usually optimized for flat extended sample geometries. In this paper, we present a laser ultrasound (LUS) method that can be used to efficiently detect defects (e.g., voids), without extensive scanning effort and without a prior knowledge of the defect location, in finite samples with curved surfaces. The developed method starts with generalized simulations of the LUS wave patterns in samples with varying radii of curvature and width as well as varying excitation size and mechanism (thermoelastic or ablative). Based on the wave pattern analysis, it is possible to predict how every point in the weld can be reached with only few excitation spots. In a second step, we assume a grid of finite size defects at locations at which such voids are most likely formed and perform a thorough simulation analysis that is based on B-Scans to find a few pairs of excitation–detection points most favorable for finding defects anywhere in the weld seam. These results are then compared to the wave pattern analysis, discussing similarities and deviations from the predictions. In a final step, the simulations are compared to experimental results, verifying the almost threefold increase in the detectability of defects by choosing the predicted optimal excitation–detection positions. It is expected that this method will significantly improve the reliability and time efficiency of detecting internal defects in samples with curved surfaces in potential industrial applications.

## Highlights

• Influence of surface curvature on the ultrasound wave field demonstrated.
• Best excitation–detection position pairs for defect detection in welds identified.
• Results of numerical investigations validated with experimental methods.

## 1 Introduction

Laser Ultrasound (LUS) is a non-contact measurement technique with a wide range of applications in materials characterization and testing. Recent advances include the non-destructive testing of composite materials and additive manufacturing components [1], [2]. The non-contact nature is a result of optical ultrasound excitation with laser pulses and optical detection of ultrasound waves at the sample surface. The most common detection methods are either based on interferometry, which is sensitive to the surface displacement of the sample, or on deflection of the reflected beam due to ultrasound-induced minute inclinations of the surface [3], [4], [5]. The ultrasonic waves are excited by absorption of energy from laser pulses at the sample surface. The resultant heating causes stresses via the thermoelastic and/or the ablative effect, which relax as ultrasonic waves [6]. Different wave modes travel into the bulk or along the surface and interact with various kinds of defects, which can be detected or localized by analyzing the back-scattered echoes.

The detectability of defects within a sample relies on the distribution of ultrasound waves, which has to cover all possible defect locations. Considering a sample with a planar surface, the distribution of the ultrasound field from a single transmitter element is dominated by the directivity pattern of the excited wave modes. For the case of LUS generation, Scruby et al. [7] and Hutchins et al. [8] showed that ultrasonic waves are much less forward directed in the thermoelastic excitation regime than in the ablative excitation regime. Krylov [9] and Yaping [10] demonstrated that thermoelastic excitation of longitudinal waves in forward direction can be enhanced by widening the excitation beam diameter and furthermore showed that in materials with a larger thermal diffusivity the radiation in forward direction also increases.

Knowledge of the distribution of ultrasound energy from the directivity pattern can be used to optimize the detection of defects in various ways. Lukacs et al. [11] could show that a combined excitation and detection sensitivity map can be used to adapt the geometry of the transmit and receive array in a LUS setup for optimizing the data acquisition speed for imaging a specific region of interest (ROI). Similarly, He et al. [12] used directivity patterns to reduce reconstruction artifacts when applying the Synthetic Aperture Focusing Technique. The directivity of laser-excited shear waves was used by Pyzik et al. [13] to optimize the distance between ultrasound excitation and detection spots for finding defects in adhesively bonded aluminum plates.

In many industrial applications, the assumption of a half-space bounded by a planar interface is not valid. An example is a weld seam, which usually has a curved surface and is confined in space. This leads to various challenges in the detection and localization of defects, such as the collection of back reflected light from the curved surface when scanning the detection laser (Lee et al. [14]). Since the transmit and receive arrays in LUS strictly follow the shape of the surface, also the imaging capability is influenced by deviations from a flat surface, which has been solved by Chen et al. [15], who adapted a Full Matrix Capture Total Focusing Technique for the purpose of detecting defects in semi-infinite curved surface samples.

The confined sample geometry also modifies the propagation of the laser-generated ultrasound. While the directivity pattern from a point source, i.e., the angular distribution of emitted shear and longitudinal waves, will not be affected by the surface curvature, such an influence has to be expected for a source of finite size. For instance, a geometrical focus is expected at the center of curvature for a wave that is emitted perpendicularly to the spherical surface. Additionally, the wave emitted into the sample can be reflected and guided by the side walls of the sample, which again influences the distribution of sound energy. The ability to find defects in structures such as weld seams is therefore not only determined by the classical directivity pattern of LUS, but rather by the wave pattern that forms inside the sample due to the effects of the specific geometry. The signals detected on the sample surface are a result of the interaction of the complex wave pattern with voids and cracks and can be used to inspect samples for defects in their volume. However, mainly as a result of the multiple reflections at the sample wall, standard reconstruction techniques such as the Full Matrix Capture Total Focusing Technique or the Synthetic Aperture Focusing Technique are difficult to apply.

Imaging of defects might not be necessary in an industrial application, where rather a quick decision based on a single parameter such as the total pore volume is required to decide whether a sample is suitable for the intended purpose. In a previous work [16] we could show that a measure of the total volume of air inclusions can be derived for non-semi-infinite samples with curved surface by analyzing an entire LUS B-Scan. The data array was measured by moving the excitation spot across the surface of a weld seam model while keeping the detection position constant. A value quantifying the variation in the received B-Scan was determined that showed a good correlation with the total volume of voids in the sample. However, taking entire B-Scans may be too time consuming and strategies have to be found to reduce the data acquisition time.

The optimization approach demonstrated in Ref. [11] provided a tenfold improvement in data acquisition speed based on the knowledge of directivity patterns for transmitting and receiving LUS waves for a predefined ROI, which was determined in a sparse scan over a flat surface. In our intended application, such an identification of an ROI is not possible due to the missing imaging capability. Instead, our goal is to find excitation–detection pairs that most likely detect the presence of defects at arbitrary, a priori unknown positions with the lowest number of measurements. We start with a generalized approach by simulating the wave patterns in samples with varying radii of curvature and width. Based on the wave pattern analysis, it is possible to predict how every point in the weld can be reached with only few excitation spots. In a second step, we assume a grid of finite size defects at locations at which such voids are most likely formed and perform a thorough simulation analysis that is based on B-Scans to find which pairs of excitation/detection points are most favorable for finding defects anywhere in the weld seam. These results are then compared to the wave pattern analysis, discussing similarities and deviations from the predictions. In a final step, the simulations are compared to experimental results.

The manuscript is organized as follows. The experimental and numerical methods are briefly presented in Section 2. A more detailed description of these methods can be found in a previous work [16]. Section 3 introduces the samples utilized for the research. Section 4 contains the results of the numerical calculation of ultrasound wave fields and the optimization of excitation–detection positions. Section 5 discusses the experimental investigations on aluminum weld seam models in order to verify the numerical investigations presented in Section 4.

## 2 Experimental and numerical methods

**Fig. 1:**
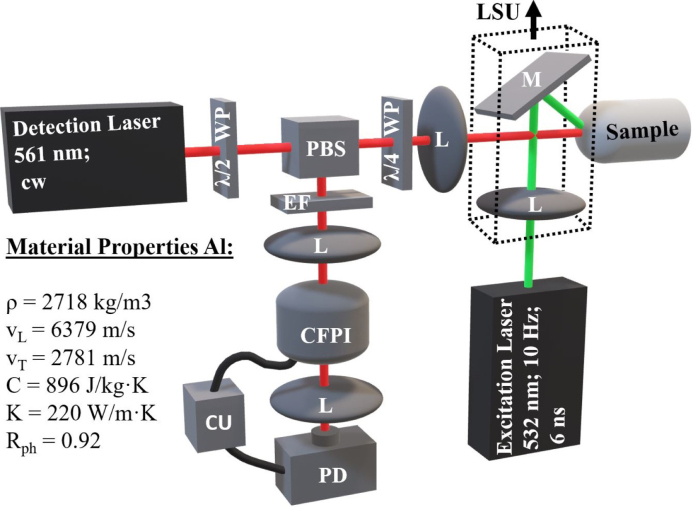
Sketch of the experimental laser ultrasound setup and list of material properties of aluminum used in the numerical simulations. PBS... Polarizing Beam Splitter, M... Mirror, CU... Control Unit, PD... Photo Diode, CFPI... Confocal Fabry–Perot Interferometer, LSU... Linear Shifting Unit, WP... Wave Plate, L... Lens, EF... Edge Filter, ρ... Density, $v_{L}$... Longitudinal Wave Velocity, $v_{T}$... Transverse Wave Velocity, C... Specific Heat Capacity, K... Thermal Conductivity, $R_{\mathrm{ph}}$... Reflectivity.

The experimental LUS setup is shown in Fig. 1. It is based on the work of Murfin et al. [17] and Dewhurst et al. [18]. A pulsed laser beam with a wavelength of 532 nm, a pulse width of 6 ns, pulse energies up to 10 mJ and a repetition rate of 10Hz was used to excite the ultrasonic waves. The optical detection system consisted of a confocal Fabry–Perot interferometer (CFPI) and a continuous wave laser beam with a wavelength of 561nm and a power of up to 170mW. The normal component of the surface velocity v, which is due to arriving ultrasonic waves, was determined by observing transient changes in the intensity of the light transmitted through the CFPI to the photodiode (PD) [18]. To actively stabilize the operating point at the half-maximum of the interference fringe, a control unit (CU) fed by the low-frequency part of the PD signal was used. This suppresses low-frequency disruptions caused by mechanical ambient vibrations and ensures high sensitivity for the higher-frequency signals. The measured and amplified signals were recorded with a detection bandwidth of 20 MHz using a digital oscilloscope and averaged over approximately 100 laser shots to increase the SNR. To further enhance the SNR, a numerical low-pass filter with a cut-off frequency of 18 MHz was employed prior to data analysis. To ensure that the detection laser was perpendicular to the sample surface, the sample was mounted on a movable sample holder that could tilt and shift in all directions. To perform a B-Scan measurement, a linear shifting unit (LSU) was used to change the position of the excitation spot on the sample surface, thereby changing the distance between excitation and detection laser spot.

In order to numerically predict the results of the experiments, thermal diffusion, thermoacoustic coupling, and elastic wave propagation were modeled in MATLAB following the work of Fellinger et al. [19], Calvo et al. [20] and Every et al. [21] and using the material parameters listed in Fig. 1. The thermal diffusion equation was solved in 1D using the finite difference method, with a spatial discretization of 98 nm and a temporal discretization of 50 ps. This values were chosen to meet the stability criteria described by Mohtar et al. [22]. As a next step, stresses were calculated from this temperature distribution using sample specific material parameters such as the density and the entries of the stiffness tensor. These stresses formed the source term of the elastodynamic equations, which were solved in 2D using the Elastodynamic Finite Integration Technique (EFIT), with acoustic attenuation being neglected. To meet the stability criteria described by Fellinger et al. [19], the spatial discretization was 6.5μm and the temporal discretization was 0.55ns. A more detailed description of these methods and a validation of the numerical model against experimental results can be found in a previous work [16]. A comparison of numerical and experimental A-Scans in a flat aluminum plate is shown in Fig. S1 in the Suppl. Mat.

**Fig. 2:**
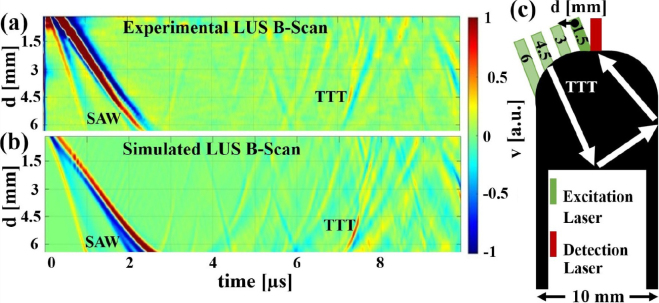
Comparison of experimental **(a)** and numerically simulated **(b)** LUS B-Scan data (particle velocity in the surface normal direction v as a function of the position of the linear shifting unit d and time) of an aluminum weld seam model with a large inclusion as depicted in the schematic illustration **(c)**. The propagation path of the two times reflected transversal wave (TTT) leading to the echo from the inclusion with the highest amplitude is indicated by the white arrows and can be seen in the provided video.

Fig. 2 demonstrates the agreement between experimental and simulated LUS B-Scans for an aluminum weld seam model. An 8 mm diameter flat bottom hole drilled from the bottom into the 20mm high weld seam model represented a large air inclusion 10mm below the surface. The unrealistically large inclusion was chosen in this demonstration to clearly separate and identify the different signal features and wave modes found in LUS experiments on this type of samples. Later simulations use more realistic inclusion sizes and positions. For the numerical simulation, the binary image of the sample on the right side of Fig. 2 was discretized. The predominant structures in the scans at early times indicate the arrival of the surface acoustic waves (SAW) at the detection laser spot. In addition, structures from direct reflections at the air inclusion and multiple reflections can be recognized. The most intense echo (TTT), arriving at approximately 7.2μs for a separation between excitation and detection spot position of d≈4.5mm, originated from a transverse wave reflected first at the air inclusion and then at the sidewall before arriving at the detection laser spot. This echo could be identified by observing the wave propagation in the numerical simulations (see Video S1), taking into account the different phase velocities of each ultrasound mode.

## 3 Samples

**Fig. 3:**
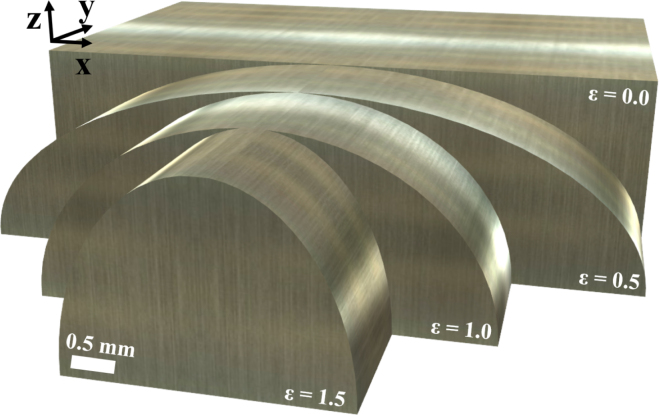
Sketches of the aluminum samples with different curvatures ɛ used in this study. For the 2D numerical investigations, the projection along y-direction of each sample was used as simulation domain.

All examined samples were made of aluminum due to its ease of fabrication and had a 2D symmetry resulting from a large extension along the y-direction. The 2D simulations therefore accurately represented the measurement setting while minimizing computational resources compared to 3D. The samples were representing weld seam models and their surface had the shape of a semi-ellipse with constant height of 2.8mm and variable curvature that changed with its width, the dimension along the x-direction. The curvature is quantified by the parameter ɛ, which is the ratio of the height of the semi-ellipse to its half width. Furthermore, to facilitate the generalization of the results, dimensionless parameters R and D were introduced for the radius of the excitation laser spot and the distance of the linear shifting unit ($R=r_{\mathrm{laser,exc}}/\mathrm{width}$ in %, D=d/width). There are also some dependencies on the absolute value of the radius of the excitation laser spot $r_{\mathrm{laser,exc}}$, which will be indicated accordingly. Fig. 3 displays sketches of four samples with different curvatures. To investigate the effect of both, curvature and reduced width on the ultrasound wave field, samples with flat surfaces (ɛ=0) of the same width as the curved ones were additionally numerically analyzed. To eliminate any effects from reflections at the lower boundary in all simulations and experiments, the samples from Fig. 3 were extended along the negative vertical direction (-z) to allow these reflections to be separated in time. Consequently, all structures visible in the B-Scans, with the exception of the SAW, originate from echoes from the inclusions. In order to find favorable excitation–detection position pairs for efficient defect detection, inclusions with a diameter of 0.5mm were introduced into the curved surface samples at 13 distinct positions (see Fig. 14). These distinct positions are representative of typical positions of defects in real welds [23], with the focus of this work being on the deeper locations in the material. While the sample sizes in the simulations were similar to anticipated sizes of weld seams, the experimental samples had to be scaled by a factor of two in order to enable the drilling of deep holes that formed the artificial inclusions. In order to achieve the same values for the R and D parameters in experiments and simulations, the size of the excitation laser spot and the scan distance of the linear shifting unit were also doubled. Since the further analysis is based on data extracted from B-Scans, we made a comparison of B-Scan simulations between samples of the original size and samples scaled by a factor of two, demonstrating an almost perfect agreement (see Fig. S2 in the Suppl. Mat.).

## 4 Numerical studies

### 4.1 Simulation of ultrasound wave fields

**Fig. 4:**
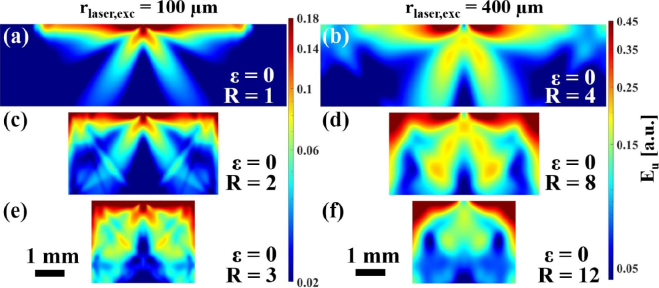
Ultrasound energy distribution $E_{u}$ in flat aluminum samples (ɛ = 0) in the thermoelastic excitation regime with central excitation (D = 0). In vertical direction the width of the samples was varied, in horizontal direction the absolute value of the radius of the excitation laser spot $r_{\mathrm{laser,exc}}$ leading to relative excitation laser spot sizes R between 1 and 12. A video of the wave propagation in **(e)** is provided in the Suppl. Mat. (Video S3).

**Fig. 5:**
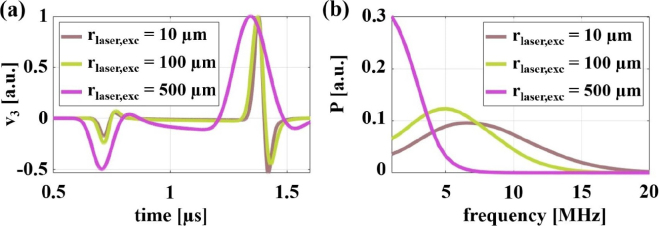
**(a)** The particle velocities in the vertical direction $v_{3}$ as a function of time and **(b)** the power spectra P are compared inside a 6.5 mm thick aluminum plate at a depth of 3.25 mm and 2.33 mm off the epicenter for varying absolute values of the excitation laser spot size $r_{\mathrm{laser,exc}}$.

The samples presented in the previous section were used for detailed LUS wave propagation simulations. As parameter study, the simulations were conducted for different excitation laser spot sizes, excitation positions and excitation regimes (thermoelastic or ablative). The laser pulse energy remained constant in all cases. To visualize the ultrasound wave field (distribution of ultrasound energy $E_{u}$), the elastic action at every tenth grid point of the simulation domain was calculated by adding up the elastic energy of all different acoustic modes over a time of 1.3μs and plotted on a logarithmic scale. The dynamic range of the color scales in the plots was set to maximize the dynamic range for the bulk waves, which resulted in the saturation of the surface waves. Fig. 4 illustrates these ultrasound wave fields in the flat samples ɛ=0. In Fig. 4(a), cones at angles of about ±30° to the surface normal can be observed, which are typical for the thermoelastic excitation regime in aluminum in the case of a small excitation spot. However, most of the energy is concentrated near the surface. Increasing the absolute size of the excitation laser spot not only increases the far field distance, but also enhances the ratio of the energy emitted into the interior of the sample to that on its surface (as can be seen e.g. when comparing Fig. 4(a) and Fig. 4(b)). This is advantageous for detecting defects inside the sample. However, due to the ultrasound being radiated at a certain angle with respect to the surface normal (especially in the thermoelastic regime), the high-frequency content of the laser-induced ultrasonic waves propagating in the material decreases with the size of the excitation laser spot (as can be seen in Fig. 5), leading to an increase in the minimum detectable defect size (≈λ/10 with λ being the ultrasonic wavelength [24]). The change in the acoustic spectrum due to the lateral spreading of the acoustic source was previously investigated by Bresse et al. [25] by comparing ultrasound waves generated by uniform and apodized radial laser beam distributions. By reducing the width of the samples (Fig. 4 (c–f)), the reflections from the left and right boundaries have an increasing influence on the way the energy is distributed. In Fig. 4 (e–f) (width ≈3.3mm), the classical directivity pattern is barely recognizable.

**Fig. 6:**
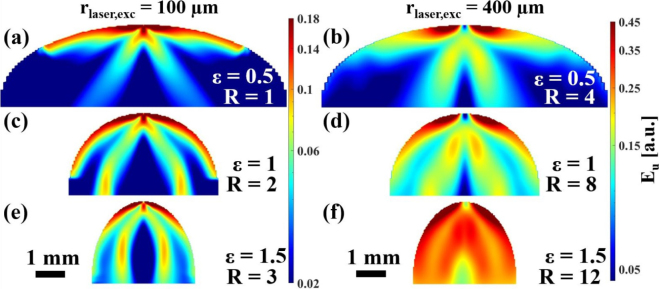
Ultrasound energy distribution $E_{u}$ in curved aluminum samples (ɛ> 0) in the thermoelastic excitation regime with central excitation (D = 0). In vertical direction the width of the samples was varied, in horizontal direction the absolute value of the radius of the excitation laser spot $r_{\mathrm{laser,exc}}$ leading to relative excitation laser spot sizes R between 1 and 12. A video of the wave propagation in **(e)** is provided in the Suppl. Mat. (Video S2).

Comparing Fig. 6 with Fig. 4, the influence of both, the decreasing sample width and surface curvature, can be observed and distinguished. At small curvatures (Fig. 6 (a–b)), the original ultrasound wave field from a flat surface were still recognizable, but with increasing curvature (Fig. 6 (c–f)), the changes in the wave pattern became quite drastic. In contrast to the flat surface, no ray-shaped reflections from the boundaries were visible in the case of the curved surface (see Fig. 6(c) and Fig. 6(e)). The energy appeared to be guided along the surface, leading to an energy distribution that follows the curved contour of the sample, especially in with the smaller absolute value of the excitation laser spot size ($r_{\mathrm{laser,exc}}=100\;\mu m$). This can also be observed by looking at the provided Video S2 showing the acoustic wave propagation for these curved surface samples and comparing it with the provided Video S3 of a flat surface. Furthermore, flat surfaces trap more energy in the upper surface region due to surface wave reflections at the top left and right corners. This effect is best seen in the case with the largest R (compare Fig. 4(f) and Fig. 6(f)).

**Fig. 7:**
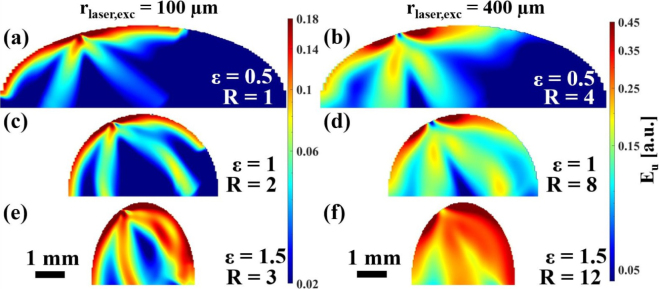
Ultrasound energy distribution $E_{u}$ in curved aluminum samples (ɛ> 0) in the thermoelastic excitation regime with off-center excitation (D = 0.24). In vertical direction the width of the samples was varied, in horizontal direction the absolute value of the radius of the excitation laser spot $r_{\mathrm{laser,exc}}$ leading to relative excitation laser spot sizes R between 1 and 12.

**Fig. 8:**
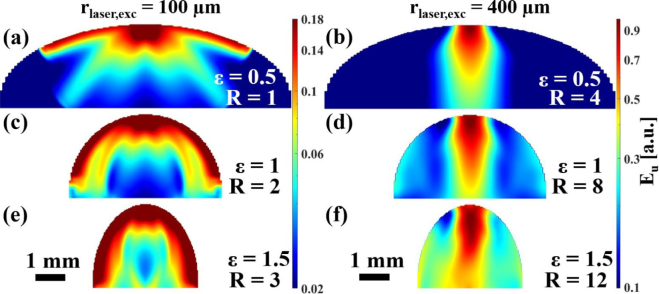
Ultrasound energy distribution $E_{u}$ in curved aluminum samples (ɛ> 0) in the ablative excitation regime with central excitation (D = 0). In vertical direction the width of the samples was varied, in horizontal direction the absolute value of the radius of the excitation laser spot $r_{\mathrm{laser,exc}}$ leading to relative excitation laser spot sizes R between 1 and 12.

The low-intensity regions in the ultrasound wave field shown in Fig. 6 are highly undesirable in material testing as they indicate regions that are not being probed for defects. The ultrasound wave field can be changed in two ways as shown in Fig. 7, Fig. 8. Fig. 7 shows the change in ultrasound wave field by changing the excitation laser spot position. Fig. 8 demonstrates the change in ultrasound wave field when an ablative laser source is used instead of a thermoelastic one. The areas of high-intensity in the ultrasound wave field have changed in both cases, compared to Fig. 6. The slight asymmetry observed in all ultrasound wave fields could be explained by the asymmetry of the experimental setup (see Fig. 1), as the excitation laser beam illuminated the sample at an angle of α≈25° with respect to the z-direction. All simulations were conducted considering the experimental conditions of oblique incidence of the excitation laser beam. This has been realized through the rotation of the coordinate system when the initial conditions are specified for the grid points in the numerical simulation.

In the ablative case shown in Fig. 8, the extreme dependence of the ultrasound wave fields on the absolute value of the excitation laser spot size $r_{\mathrm{laser,exc}}$ was notable. In all previous cases, the energy in the transverse wave always outweighed the energy in the longitudinal wave. In the ablative case with a large $r_{\mathrm{laser,exc}}$, this ratio changed and the forward directed radiation of the longitudinal waves became dominant. It is also shown in Fig. 8, that in the ablative case with $r_{\mathrm{laser,exc}}=400\;\mu m$, the ultrasonic field was focused towards the geometrical focal point of the sample surface. In addition, in ablative excitation, it was more pronounced that the increase of the absolute laser spot size caused more energy to be radiated into the interior of the sample, compared to the energy that propagated along the surface.

### 4.2 Favorable laser spot positions

In the following sections, the discussion is limited to the thermoelastic case, since the experimental detection of ultrasonic waves in reflection mode (when excitation and detection laser spot are close together) at extreme ablative excitation was difficult due to the disturbance by the ultrasound propagating in air.

Based on the results of the ultrasound wave field analysis, the next step towards an efficient inspection system was to find the best pairing of excitation–detection laser spots. Since the ultrasound wave fields shown in the previous section have low-intensity regions, several pairs will be needed, especially for inspecting the entire volume of large samples. As demonstrated in the previous section, increasing the absolute excitation spot size leads to more elastic energy being radiated into the sample interior, resulting in smaller low-intensity regions in the sample volume. Therefore, only the larger absolute spot size of 400μm will be considered in the following, leading to relative excitation laser spot sizes R of 4, 8 and 12 for the different samples. It should also be noted that for all excitation positions considered in this numerical study, at least a small amount of backscattered energy from the inclusions reached each of the detection points. Therefore, this study focused on finding the pair of excitation–detection position where the most energy was backscattered from the inclusions in order to improve the SNR in experimental weld seam inspections.

**Fig. 9:**
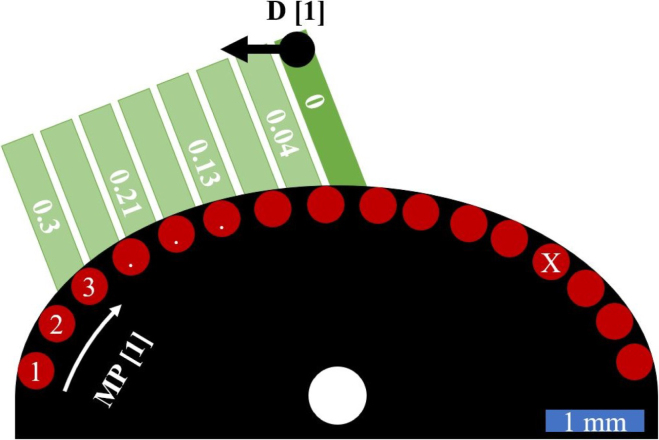
Sketch of different excitation D and detection MP laser spot positions for the numerical investigation of favorable positions to detect air inclusions in weld seam models.

In order to find these best excitation–detection position pairs, simulations were carried out with multiple inclusion positions distributed on a grid within the sample volume. The air inclusions were assumed to have a circular shape with a diameter of approximately 0.5mm. In addition, the position of the excitation laser spot D and the width of the sample were varied. For each simulation, the displacement of the sample surface was recorded at many points along the surface. Fig. 9 shows the numerical setting using the example of the aluminum weld seam model with the maximum width (ɛ=0.5) and an inclusion in the center. The green lines represent the different excitation laser spot positions D for the individual simulations, the red points correspond to different measurement/detection laser spot positions (MP) on the surface for which the displacement was recorded in all simulations. Each simulation with specific values of excitation laser spot position, sample width and inclusion position was conducted twice, once with and once without the inclusion. The displacement signals recorded for both cases were subtracted to calculate the elastic energy backscattered from the inclusion (absolute square value). The energy at each individual detection point was summed up over a time of 4μs to obtain a measure (E-value in units of a physical action) indicating the suitability of that point for detecting air inclusions in the simulated sample under the given excitation conditions. The backscattered energy was analyzed as there is a demonstrated correlation between the E-value (sum of the squared absolute values) in a B-Scan of a weld seam model and its total volume of process pores [16].

**Fig. 10:**
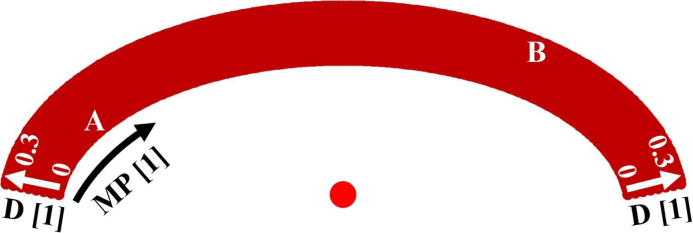
Explanation of the 2D representation of the numerical results. In the azimuthal direction the different detection laser spot positions MP from Fig. 9 are plotted. In the radial direction the different excitation laser spot positions D from Fig. 9. The point A for example represents the excitation position D = 0 and the measurement position MP = 3 from Fig. 9 and the point B represents D = 0.3 and MP = X from Fig. 9.

To analyze the suitability of excitation–detection pairings, the calculated E-values are plotted in a 2D intensity plot with varying detection positions MP on one axis and varying excitation positions D on the other axis. In order to be able to better interpret the different detection positions, they are displayed in the shape of the sample surface, so that the excitation position varies in the radial direction. In Fig. 10 this 2D representation of pairs of excitation–detection laser spot positions is explained.

**Fig. 11:**
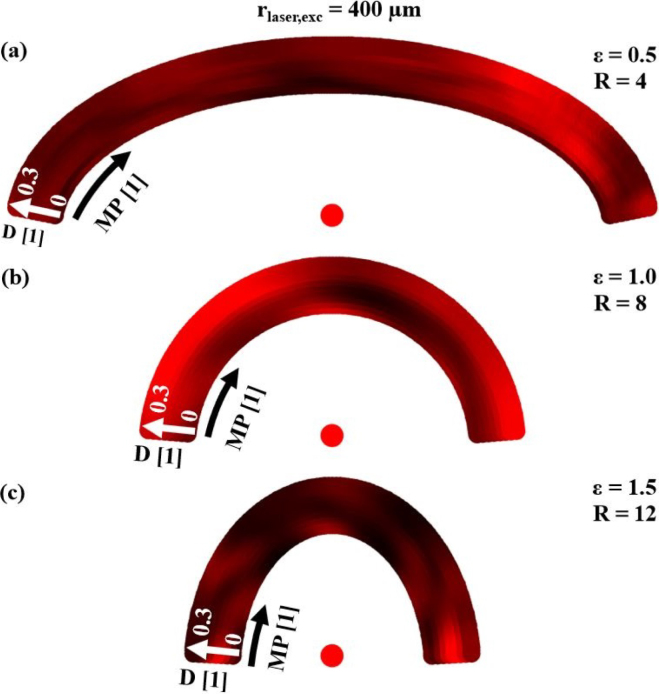
2D representation (explained in Fig. 10) of the best pair of excitation–detection positions pairs for detecting a defect in the center of weld seam models with different widths ɛ. The absolute value of the excitation laser spot size $r_{\mathrm{laser,exc}}$ was 400 μm leading to relative excitation laser spot sizes R between 4 and 12. In all three cases **(a)**, **(b)** and **(c)** the excitation position D varies in radial direction and the detection position MP varies in azimuthal direction.

An example of this representation is shown in Fig. 11 for a weld seam model with an inclusion in the center. The best excitation–detection position pairs correspond to the high-intensity regions in this 2D representation. For the given test case in Fig. 11(a) with ɛ=0.5, it is recommended to choose the excitation position with D = 0.3 from Fig. 9 (the outermost arc in the 2D representation) and the detection position MP=X from Fig. 9 to efficiently detect this defect in the center of the weld seam model. The asymmetry observed in this 2D representation is a consequence of the fact that only one half of the weld seam model has been considered for ultrasound excitation (see Fig. 9). Excitation on the other (right-hand) half of the sample would lead to a mirrored distribution.

**Fig. 12:**
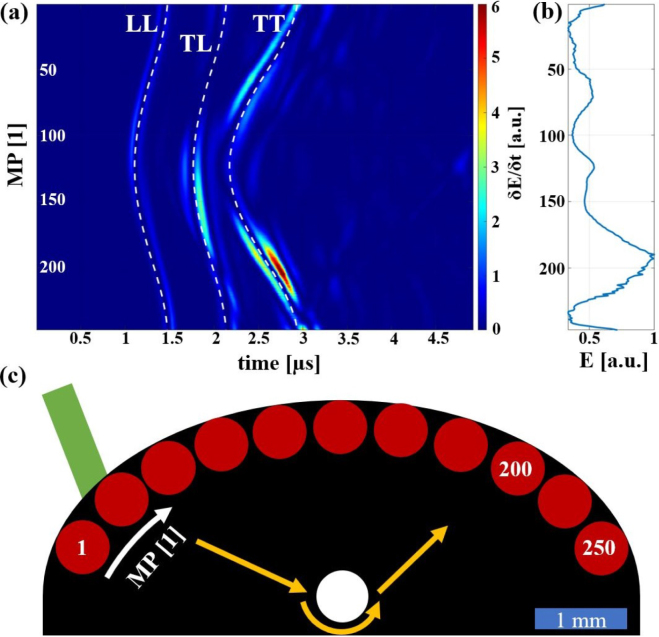
Illustration of the best detection position for a large weld seam model (ɛ=0.5 and R = 4) with an inclusion in the center using the excitation position D = 0.3 as depicted in the schematic illustration **(c)**. **(a)** illustrates the temporal derivative of the E-value δE/δt for the various measurement positions MP, with the distinct echoes from the inclusion labeled as LL, TL, and TT. **(b)** depicts the E-value E for the different MP. The yellow arrows in **(c)** indicate the path of the echo arriving at the MP slightly after the TT echo.

Fig. 12 illustrates why this detection position is the best choice. The intensity plot in Fig. 12(a) illustrates the temporal variation of the E-value (δE/δt in units of a physical energy) for the excitation position at D = 0.3. Fig. 12(b) displays the E-value for each MP, which corresponds to the summation over all times in the intensity plot. The y-axis represents the MP ranging from 1 to 250. Fig. 12(c) depicts this setting. It can be seen that in the region of MP=200 (which corresponds to MP=X in Fig. 9) the E-value has its maximum, being more than twice as high in amplitude as the minimum. The calculated theoretical arrival times of the echoes from the inclusion were used to assign the echoes to the structures in the intensity plot. LL corresponds to the echo from the longitudinal wave, which has the lowest intensity and is approximately of equal strength at all MP. TL corresponds to the mode-converted echo (transverse to inclusion, longitudinal to detection), which has its maximum at the central MP. The dominant structure, however, originates from the transverse echo TT, which reaches its highest intensity at the off-center MP. In the area around MP=200, the TT echo also exhibits constructive interference with another echo that originates from a skimming surface wave that travels around the inclusion. The pathway of this second echo is indicated by the golden arrows in Fig. 12(c). The structure corresponding to this echo can also be observed at other MP shortly after the TT echo.

**Fig. 13:**
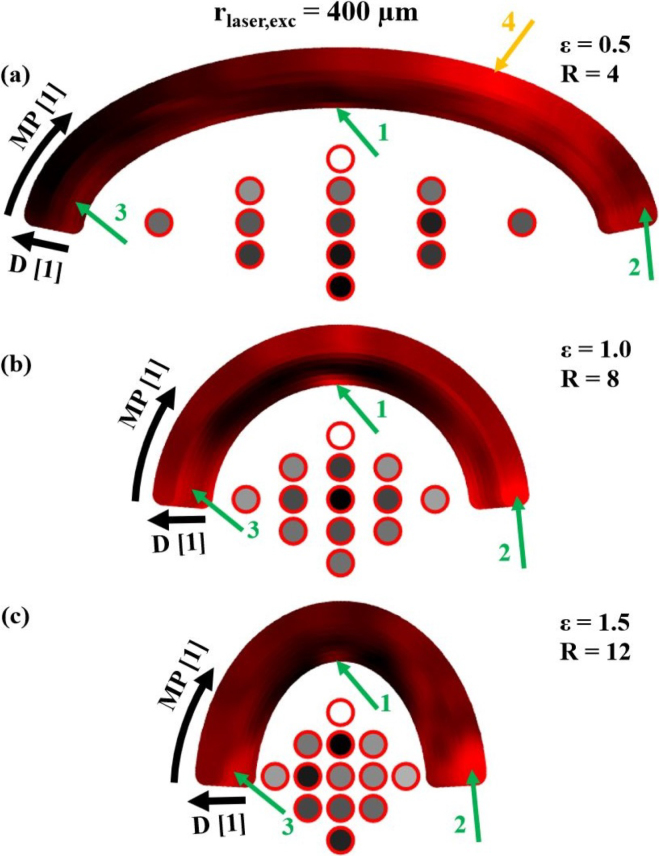
2D representation (explained in Fig. 10) of the best excitation–detection position pairs summed over all different inclusions for three different samples with different curvatures (**(a)**ɛ=0.5, R = 4; **(b)**ɛ=1, R = 8 and **(c)**ɛ=1.5, R = 12). In all three cases **(a)**, **(b)** and **(c)**D corresponds to the excitation position (radial axis), MP corresponds to the detection position (azimuthal axis) and $r_{\mathrm{laser,exc}}$ was 400μm. The best positions for all inclusions and ɛ were marked with arrows and numbers. The grey level of the inclusion indicates how well it is detectable compared with the others.

**Fig. 14:**
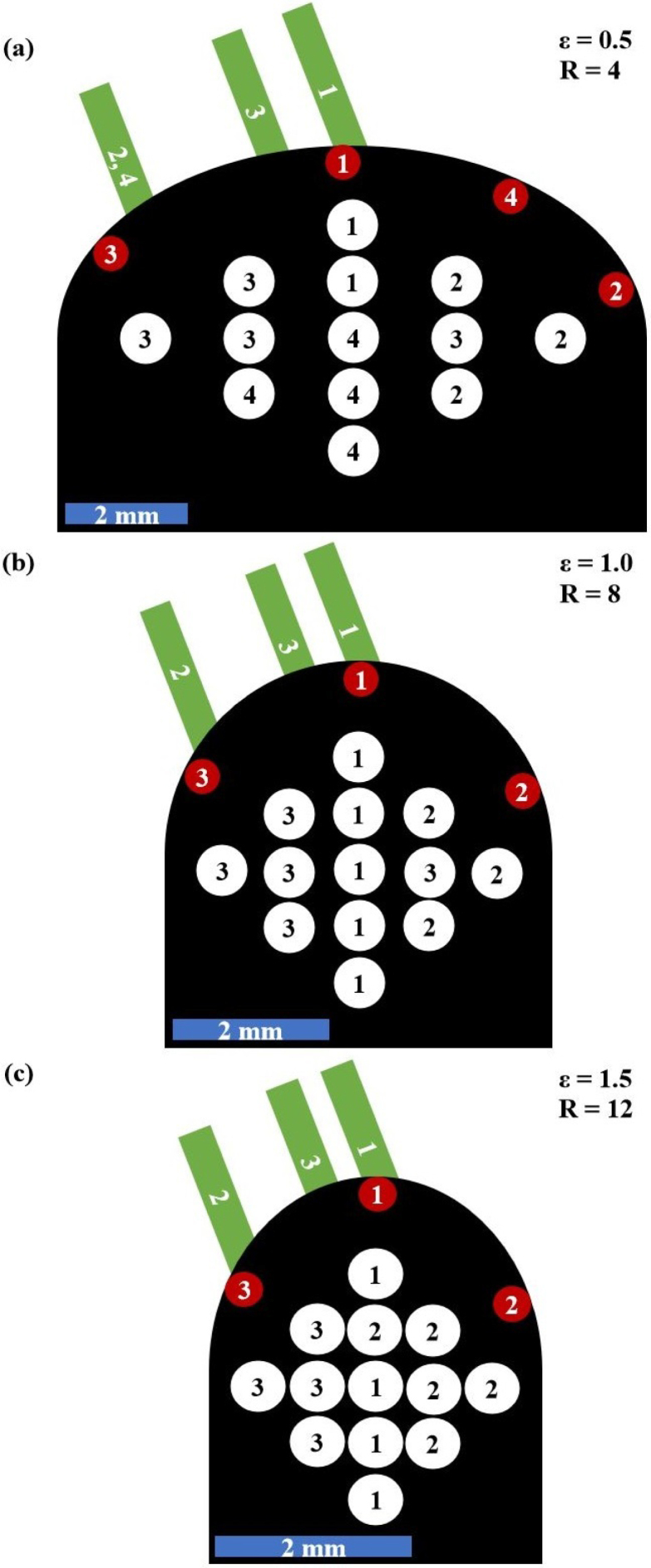
Schematic drawing of the best excitation–detection position pairs found in Fig. 13 using an absolute excitation laser radius of 400μm, leading to relative excitation laser spot sizes R between 4 and 12 for the different curvatures ɛ. In all three cases **(a)**, **(b)** and **(c)** the green lines correspond to the excitation positions D and the red points to the detection positions MP. The number labeling each inclusion refers to the pair of excitation–detection positions best suited for detecting that inclusion.

Fig. 13 shows the sum over all 2D representations of the different inclusions in the weld seam models. Each inclusion was simulated separately and its 2D representation was normalized to one prior to the summation. The value of this normalization factor is represented by the brightness of the face of each inclusion and describes how well an inclusion can be detected compared to the other inclusions. As expected, the top inclusion is among the easiest to detect in all weld seam models of different widths. It is also worth noting that inclusions situated in geometric focal points of the surfaces are the darkest, thus the most difficult to detect in the thermoelastic case. Comparing the results of the three different widths, three pairs of excitation–detection positions can be identified (green arrows), which in all cases were particularly well suited for testing the weld seam models for defects. In the case with ɛ=0.5, i.e. for large weld seam models, a fourth pair (orange arrow) was added. These pairs are also shown in Fig. 14 for the different samples and marked there with the same numbers.

Furthermore, each inclusion is labeled with the number of the excitation–detection pair most suitable for detecting that inclusion. For the large sample, detection position 4 was particularly suitable for deep inclusions. Moreover, with few exceptions, the inclusions could be easily categorized following their favorable detection position as left, center or right.

**Fig. 15:**
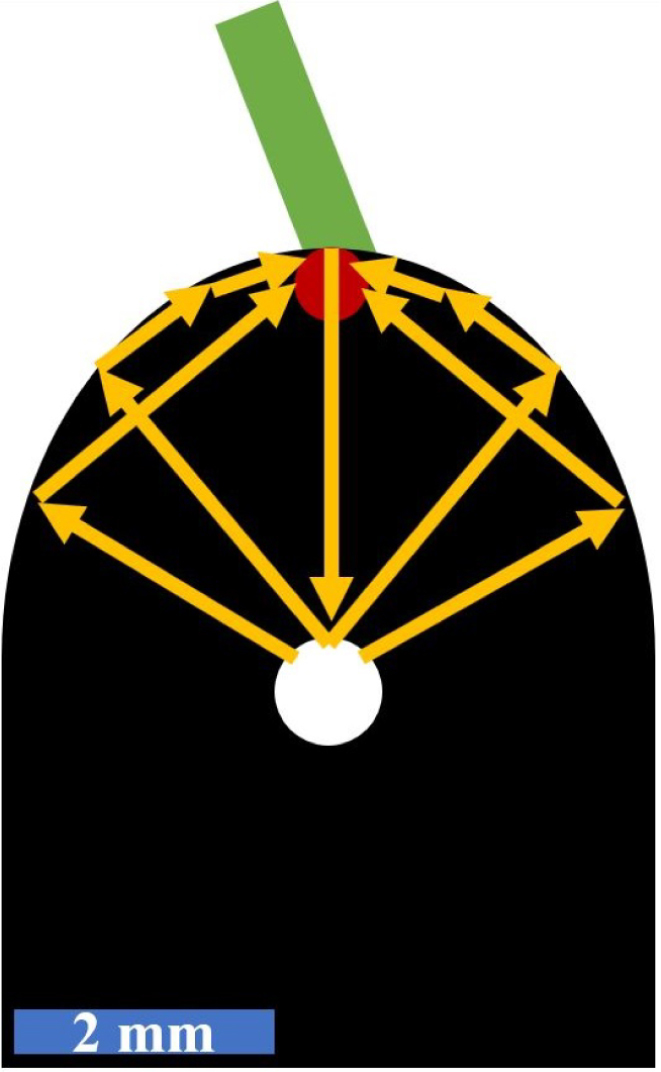
Schematic sketch of the ultrasound wave paths (yellow arrows) for excitation–detection pair number one in the case of an air inclusion in the center of the weld seam model, demonstrating the suitability for detecting this kind of defect.

Considering just the directivity and the ultrasound wave field pattern, it is not obvious why the excitation–detection pair number one is particularly well suited for detecting inclusions in the center. Fig. 15 illustrates the typical path of the received echoes. The inclusion scatters the waves to both sides, which then return to the detection spot as either bulk or surface waves. These waves from both sides interfere constructively at the detection point, due to the symmetry of the sample, the inclusion, and the excitation conditions. Despite the low energy radiated along the center line and reaching the inclusion, this interference produces a strong signal.

The favorable excitation–detection pairs two and three can be explained by the ultrasound wave fields and the close distance of the detection laser spot to the respective inclusions. Additionally, the cavity formed by the two parallel boundary surfaces at the lower end of the half-ellipse enhances the detectability of multiple reflections, favoring detection in this region.

Further favorable excitation–detection position pairs could be found by simply mirroring the previous ones.

## 5 Experimental verification

The following section presents a verification of the numerical results obtained in the previous section. As mentioned above, all dimensions had to be doubled for the experimental samples, including the absolute value of the excitation laser spot size $r_{\mathrm{laser,exc}}$ and the position of the linear shifting unit d, so that the dimensionless parameters R and D were kept constant. In Fig. S3 of the Suppl. Mat., energy value profiles like in Fig. 12(b) were calculated numerically for the same size as the experimental samples (all dimensions doubled) and compared to the ones used in all the numerical simulations, demonstrating the close agreement.

**Fig. 16:**
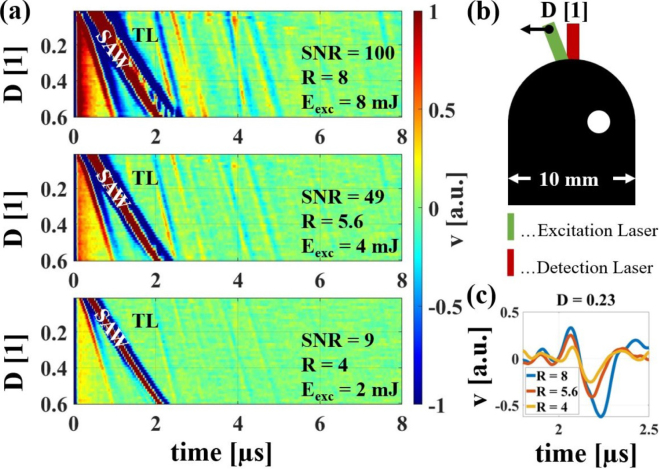
**(a)** Comparison of SNR of B-Scans obtained with varying laser spot sizes R from a 10 mm wide weld seam model with a 1.8 mm diameter air inclusion off-center, sketched in **(b)**. All B-Scan images contain structures from surface acoustic waves (SAW) and structures from echoes, e.g., TL. **(c)** shows the TL echo in A-Scans of the different excitation laser spot sizes at D = 0.23. The pulse duration of the echo indicates the change in the ultrasound spectrum. D... excitation position; v... particle velocity in normal direction $E_{\mathrm{exc}}$... excitation laser energy.

As stated in Section 4.1, when detecting inclusions that are not too small compared with the ultrasonic wavelength λ, it is beneficial to use a larger excitation laser spot, as this increases the amount of energy radiated into the sample’s interior. This is confirmed by B-Scans conducted on the sample depicted in Fig. 16(b), using three different relative excitation laser spot sizes R (8, 5.6, and 4). Note that the extension of the sample in vertical direction is larger than displayed in Fig. 16(b) to avoid echoes from the lower boundary. Therefore, the visible echoes are all originating at the displayed inclusion. The structures of the echoes are determined by the curved sample surface, which has an influence on the path between excitation and detection points along the inclusion and also causes a nonlinear relation between the horizontal shift D of the linear shifting unit and the position of the excitation spot. In a previous work, the theoretical arrival times of the respective echoes were calculated geometrically and compared with the structures visible in the B-Scans for samples with different curved surfaces and different inclusion positions [16]. The excitation laser energy density was kept constant for all three cases staying within the thermoelastic range. Fig. 16(a) displays three B-Scans, illustrating the decrease in SNR of the TL echo (from 100 to 9) as the size of the excitation laser spot decreases from top to bottom. The TL-echo was chosen for analysis given that it is the echo with the greatest displacement in the surface normal direction. Additionally, broader structures for surface waves (SAW) and echoes from the inclusion (as depicted for the TL-echo in Fig. 16(c)) were observed as the excitation laser spot size increased, indicating a shift of the ultrasound spectrum towards larger wavelengths (see Fig. 5 for comparison).

**Fig. 17:**
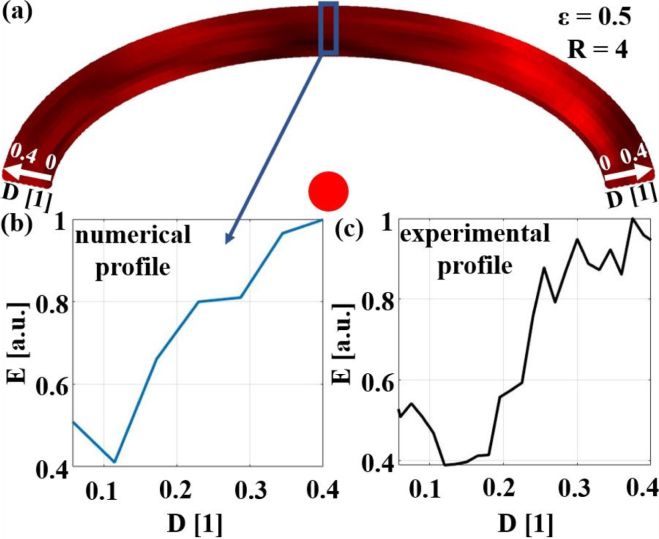
Comparison of experimentally measured E-values E with a fixed detection position at the center of the sample and varying excitation positions D, depicted in **(c)**, and a radial profile through the numerical 2D representation **(a)**, depicted in **(b)**, of a sample with ɛ=0.5 and an inclusion in the center. The radius of the excitation laser spot was R = 4.

**Fig. 18:**
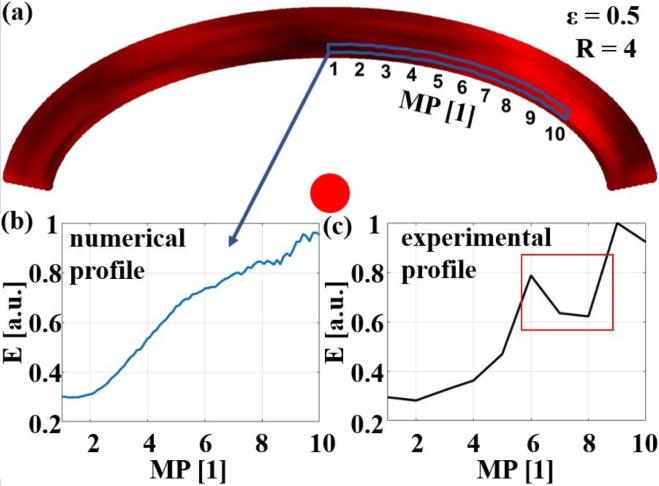
Comparison of experimentally measured E-values E with a fixed excitation position (D = 0.04) and varying detection positions MP, depicted in **(c)**, and an azimuthal profile through the numerical 2D representation **(a)**, depicted in **(b)**, of a sample with ɛ=0.5 and an inclusion in the center. The radius of the excitation laser spot was R = 4.

To validate the results of the numerical study for the best pairs of excitation–detection spots for the weld seam model with the largest width (ɛ=0.5), two different experiments were conducted and compared with the numerical results (see Fig. 17, Fig. 18). For the first experiment, shown in Fig. 17, the detection laser spot was fixed at the center of the weld seam model and the position of the excitation laser spot was varied between D = 0 and D = 0.4 by using the linear shifting unit. For each excitation laser spot position, the E-value of the signal excluding surface waves was calculated. Plotting this value over the position of the excitation laser spot (shown in Fig. 17(c)) corresponded to a profile through the numerical 2D representation in the radial direction (shown in Fig. 17(b)). Both profiles were normalized to their maximum values and are in good agreement, showing a twofold increase in the E-value by choosing the optimum excitation position.

The result of the second experiment is depicted in Fig. 18. In this case the excitation position was kept fixed and the detection position was varied. Again, the numerical and an experimental profile are in good agreement, showing an almost threefold increase in the E-value by choosing the optimum detection position. The drop in the energy value around the seventh and eighth detection positions (marked with the red box) could be explained by the fact that in this region the main echo from the inclusion overlapped with the surface wave, which was excluded in the E-value calculation.

**Fig. 19:**
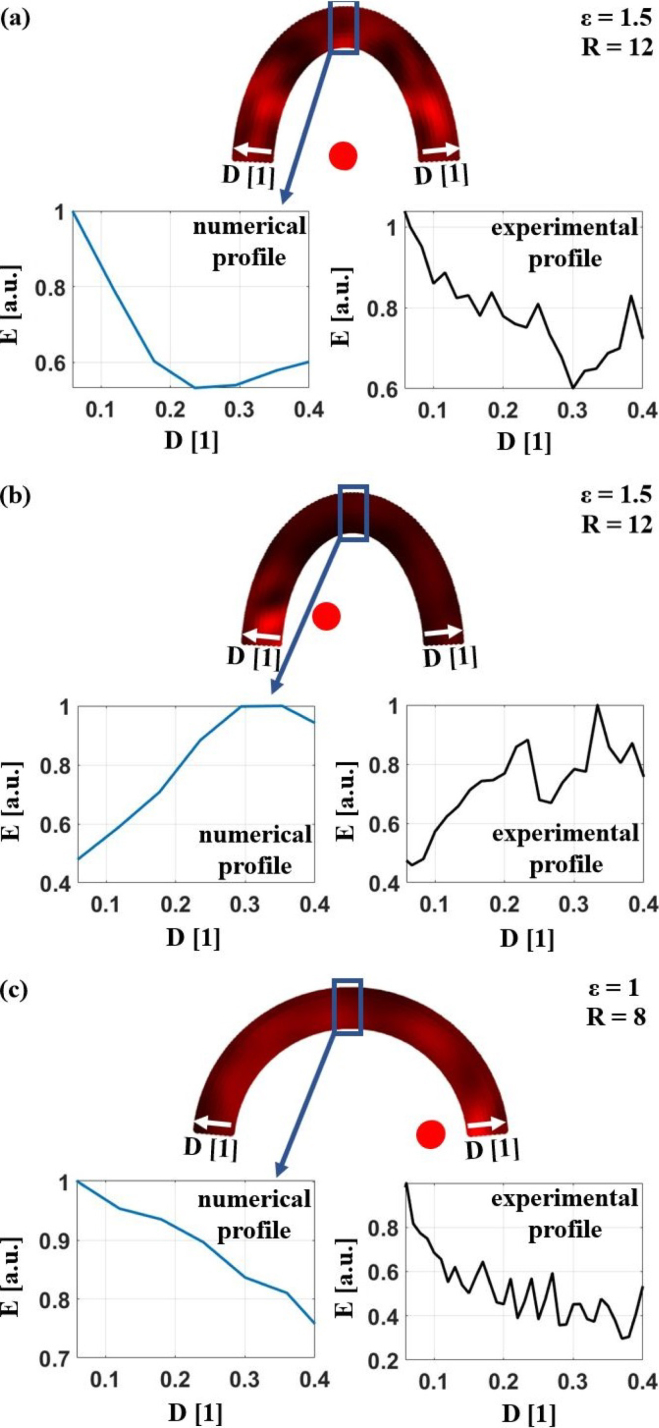
Comparison of experimentally measured E-values E with a fixed detection position (at the center of the sample) and varying excitation positions D with radial profiles through the numerical 2D representation like in Fig. 17 or three different samples with different curvature and inclusion positions (**(a)**ɛ=1.5, R = 12; **(b)**ɛ=1.5, R = 12 and **(c)**ɛ=1, R = 8). R... relative excitation laser spot size.

Fig. 19 shows further comparisons between experimental and numerical radial profiles for samples with different ɛ and inclusion positions. The agreement between the numerical and experimental profiles is satisfactory in all cases, showing a twofold increase in the E-value by choosing the optimum excitation position.

## 6 Conclusion and outlook

Laser ultrasonic testing of samples such as weld seams has to consider the surface curvature and the limited size of the investigated object, both having a strong impact on the ultrasound distribution.

It could be demonstrated that the excitation laser spot size and the location of excitation and detection on the sample surface are factors that can be optimized for rapid screening for potential defects without acquisition of complete B-Scans and without a priori knowledge of the defect locations. Our findings are based on a general analysis of the acoustic wave distribution in a variety of sample geometries as a function of excitation spot size and position, which can be used to predict favorable excitation strategies. Furthermore, in a second step a thorough analysis that included the interaction of the LUS field with finite-size defects demonstrated that optimization of excitation–detection pairs also has to include multiple reflections at the sample boundaries.

Numerical simulations showed that with increasing curvature the ultrasound field is generally more confined by the boundaries. It could be distinguished from a similar effect observed by reducing the width of flat surface samples. It was concluded that a large excitation laser spot R is preferable to a small one for detecting defects that are not significantly smaller than the ultrasonic wavelength. Low-intensity regions in the ultrasound wave field were discovered and methods to change the energy distribution to radiate more energy into these regions were discussed. A numerical model considering a raster of finite-sized voids was then used to find the most suitable pairs of excitation–detection laser spot positions for the most efficient testing of air inclusions in arbitrarily convex curved aluminum weld seam models. By selecting the optimal excitation–detection pairings, it was shown that an almost threefold increase in the E-value can be achieved, while the number of measurement points can be decreased, thereby enhancing time efficiency. These findings in the thermoelastic regime were verified with experiments. Furthermore, it has been discovered that the thermoelastic and ablative excitation regimes complement each other perfectly for detecting defects in arbitrarily convex curved surfaces. In the thermoelastic regime, the geometrical focal point of the sample surface represents a kind of blind spot, while in the ablative regime the wave field is focused on it.

In the future, the methodology will also be investigated for the quality assurance of other materials. It should be noted that slight variations of the results are to be expected, as the directivity pattern depends on the material parameters. Furthermore, more complex topographical sample structures will be examined in order to cover a variety of scenarios in potential scientific and industrial applications.

## CRediT authorship contribution statement

**Markus Saurer:** Writing – original draft, Visualization, Software, Methodology, Investigation, Formal analysis, Data curation. **Guenther Paltauf:** Writing – review & editing, Supervision, Methodology, Investigation, Conceptualization. **Robert Nuster:** Writing – review & editing, Validation, Supervision, Resources, Project administration, Methodology, Funding acquisition, Formal analysis, Conceptualization.

## Declaration of competing interest

The authors declare that they have no known competing financial interests or personal relationships that could have appeared to influence the work reported in this paper.

## Data Availability

Data will be made available on request.

## Appendix A Supplementary data

Video S1 Video S1. Wave propagation in the sample used for the comparison between experimental and numerical B-Scans. The arrows indicate the path of the echo with the highest amplitude.

Video S2 Video S2. Simulation of wave propagation in a sample with a curved surface, used for calculating the laser-excited energy distribution in such a sample.

Video S3 Video S3. Simulation of wave propagation in a sample with a flat surface, used for calculating the laser-excited energy distribution in such a sample.

MMC S4 Fig. S1, Fig. S2 and Fig. S3 from Suppl. Mat..
