# Supplementary material for: Laser ultrasound wave pattern analysis for efficient defect detection in samples with curved surfaces
Source: Photoacoustics. 2024 Sep 27;40:100654. doi: 10.1016/j.pacs.2024.100654 (PMC11483478; doi:10.1016/j.pacs.2024.100654)
Supplement: MMC S4 — Fig. S1, Fig. S2 and Fig. S3 from Suppl. Mat.. [file mmc4.pdf]

Supplementary Material:

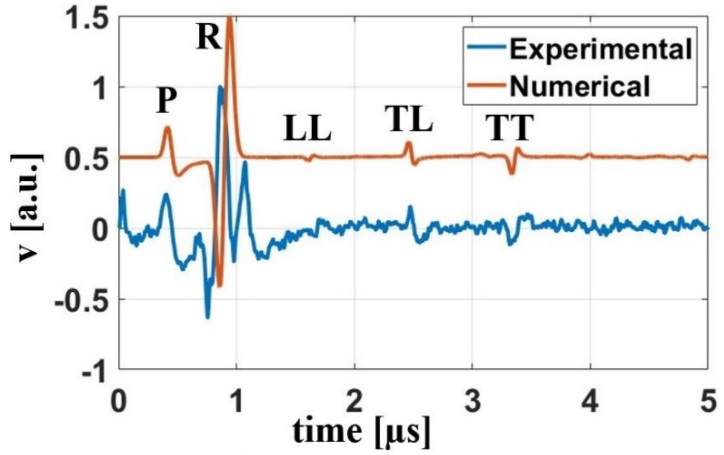

Fig. S1: Comparison of experimental and 2D numerically simulated A-Scans (normal velocity v as a function of time) at a distance between excitation and detection of about 2.5 mm on a flat, 5 mm thick, aluminum plate. Apart from the different shape of the Raileigh wave (R) all other peaks (P, LL, TL, TT) are nicely reproduced by the numerical simulation in both polarity and amplitude. The different shape of the surface wave can be explained with the fact that the excitation conditions (excitation spot shape and pulse duration) are not accurately given in the numerical simulations due to the fact that they have not been measured accurately and in order to reduce the numerical effort.

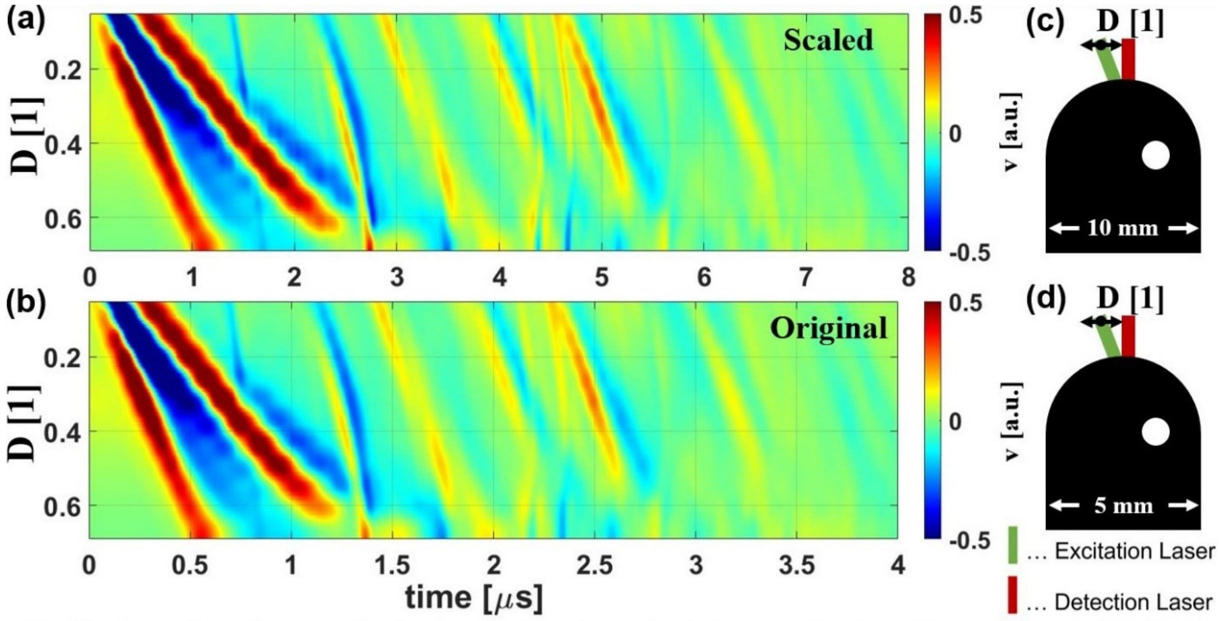

Fig. S2: Comparison of numerically simulated B-Scans (normal velocity v as a function of the excitation position D and time) for a weld seam model with an inclusion off center ((**b**) and (**d**)) with a B-Scan of weld seam model where all dimensions, including the excitation laser spot size, have been doubled ((**a**) and (**c**)). Both B-Scans (**a**) and (**b**) are in good agreement.

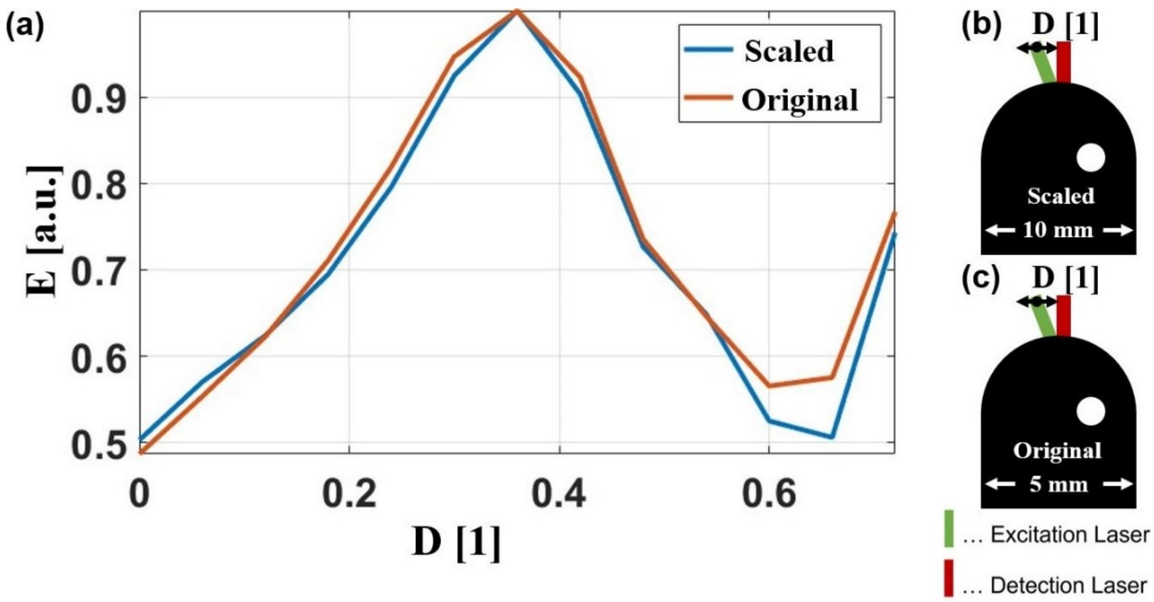

Fig. S3: Comparison of a numerically simulated energy profile (E-value as a function of excitation position D) for a weld seam model with an inclusion off center (Original) depicted in **(c)** and one where all dimensions, including the excitation laser spot size, have been doubled (Scaled) depicted in **(b)**. The tendencies of the Original profile are satisfactorily reproduced by the Scaled profile (see **(a)**).
